# No Excess Mortality in Patients Aged 50 Years and Older Who Received Treatment for Ductal Carcinoma *In Situ* of the Breast

**DOI:** 10.1155/2012/567506

**Published:** 2012-05-13

**Authors:** Esther Bastiaannet, Willemien van de Water, Rudi G. J. Westendorp, Maryska L. G. Janssen-Heijnen, Cornelis J. H. van de Velde, Anton J. M. de Craen, Gerrit-Jan Liefers

**Affiliations:** ^1^Department of Surgery, Leiden University Medical Center, P.O. Box 9600, 2300 RC Leiden, The Netherlands; ^2^Department of Gerontology and Geriatrics, Leiden University Medical Center, 2300 RC Leiden, The Netherlands; ^3^Department of Clinical Epidemiology, VieCuri Medical Centre, 5912 BL Venlo, The Netherlands; ^4^Department of Research, Eindhoven Cancer Registry, 5612 HZ Eindhoven, The Netherlands

## Abstract

*Background*. The incidence of ductal carcinoma *in situ* (DCIS) has increased at a fast rate.The aim of this study was to assess the incidence and treatment in the Netherlands and estimate the excess mortality risk of DCIS. *Methods*. From the Netherlands Cancer Registry, adult female patients (diagnosed 1997–2005) with DCIS were selected. Treatment was described according to age. Relative mortality at 10 years of follow-up was calculated by dividing observed mortality over expected mortality. Expected mortality was calculated using the matched Dutch general population. *Results*. Overall, 8421 patients were included in this study. For patients aged 50–64, and 65–74 an increase in breast-conserving surgery was observed over time (*P* < 0.001). For patients over 75 years of age, 8.0% did not undergo surgery; this percentage remained stable over time (*P* = 0.07). Overall, treated patients aged >50 years experienced no excess mortality regardless of treatment (relative mortality 1.0). *Conclusion*. The present population-based study of almost 8500 patients showed no excess mortality in surgically treated women over 50 years with DCIS.

## 1. Introduction

Carcinoma *in situ* of the breast is defined as abnormal proliferation of epithelial cells that do not trespass the basal membrane of the breast ductal or lobular system and consist of a heterogeneous group with different types of histology and also different prognosis [1]. The incidence of ductal carcinoma *in situ *(DCIS) has increased significantly in all parts of the world including the Netherlands, mainly due to the introduction of breast cancer screening. The biologic behavior of DCIS detected by mammography is unclear [2]. Few treated patients will ultimately die of breast cancer; however, despite the relatively benign nature of DCIS, patients commonly undergo mastectomy [2–4]. The risks of overdiagnosis and overtreatment have been discussed in several studies [2, 3, 5]. Nonetheless, some patients with DCIS have a less benign course than other patients, and it is still not possible to identify which DCIS lesions will progress to invasive carcinoma and in what time interval [6]. Besides, although DCIS is thought of as an early-stage cancer, lesions can be quite large [6].

Most clinical series have focused on the risk of breast cancer recurrence, rather than risk of death per se [3]. Population-based reports of actual deaths from breast cancer in women with DCIS are scarce, but show little excess mortality [7, 8]. The mass mammographic screening program in the Netherlands started in 1990/1991 for females aged 50–70 years; in 1997 the upper age limit of the screening program was increased to 75 years. The aim of this study was to assess the incidence and treatment of patients with DCIS in the Netherlands from 1997 to 2005 and to calculate the number of observed deaths versus the number of expected deaths based on the general population to estimate the excess mortality risk of patients diagnosed with DCIS.

## 2. Methods

### 2.1. Study Population

PALGA, the nationwide Dutch network and registry of histo- and cytopathology, regularly submits reports of all diagnosed malignancies to the regional cancer registries. The national hospital discharge databank, which receives discharge diagnoses of admitted patients from all Dutch hospitals, completes case ascertainment. Trained cancer registry personnel collect data on diagnosis, staging, and treatment from the medical records, including pathology and surgery reports, using the registration and coding manual of the Dutch Association of Comprehensive Cancer Centers. All data from the regional cancer registries are merged into the Netherlands Cancer Registry (NCR). From the NCR database, adult female patients with DCIS diagnosed between 1997 and 2005 were selected (*n* = 8421). Patients with a history of other malignancies were excluded. Histopathology was according to the national protocols in the Netherlands (http://www.oncoline.nl/); central review of the histopathology was not performed. DCIS was defined according to these protocols, and microinvasion (T1mi) was not included.

### 2.2. Statistical Analysis

Age was divided into younger than 50, 50–64, 65–75, and 75 years and older where the first and last groups were not invited for screening in the selected period. Treatment was assessed and stratified for age. Changes over time in treatment were studied using chi-square tests or linear regression analysis. Vital status was established directly from the patient's medical record or, in case of missing values through linkage of cancer registry data with the municipal population registries which record information on their inhabitant's vital status. As cause of death is not known in these cancer registry data, we used relative mortality. Relative mortality for 10 years of follow-up in the cohort was calculated by dividing observed mortality in the cohort at 10 years and expected mortality. Expected mortality was estimated based on the corresponding (age, sex, and year) general population (national life tables). National life tables were obtained from Statistics Netherlands. Mortality was stratified for age (to assess differences in young and elderly patients) and treatment. The aim of the stratification in treatments was not to compare treatments between strata as this would not be possible due to confounding by indication. Instead, we aimed to assess excess mortality over strata for each surgical treatment group.

## 3. Results

**Figure 1:**
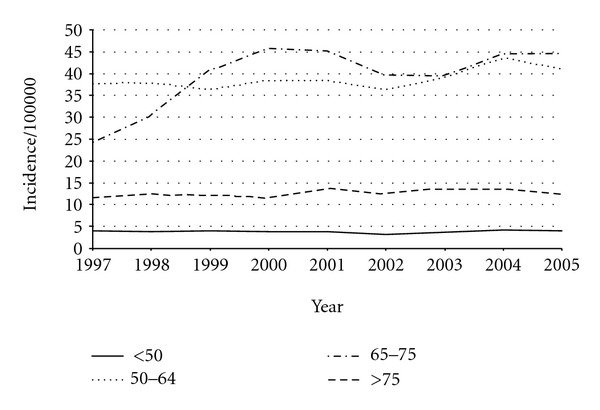
Incidence per 100 000 of DCIS in the Netherlands.

Overall, 8421 patients with DCIS were included in this study.  Table 1 shows the characteristics of the study population. Overall, almost half of all DCIS was diagnosed in patients aged 50–64 years.  Figure 1 shows the incidence per 100 000 in the Netherlands over the period 1997–2005. The incidence for patients under 50 years remained stable (range 3.1–4.1 per 100 000). Incidence for patients aged 75 years and older remained stable around 12 per 100 000 (range 11.7–13.7 per 100 000). The incidence slightly increased for those aged 50–64 years from 37.8 to 41.0 per 100 000 and almost doubled for the age group 66–74 from 24.4 to 44.6 per 100 000.

Table 2 shows the treatment for patients with DCIS in the Netherlands according to age and period of diagnosis. Patients younger than 50 years often underwent mastectomy (range 55.7% to 60.5%) or breast-conserving surgery (range 38.9% to 46.3%). Over all the years, only 0.6% did not undergo surgery. Patients aged 50–64 and 65–74 more often underwent breast-conserving surgery, and this proportion significantly increased over time (*P* < 0.001). For the elderly patients ≥75 of age, 7.6% did not undergo surgery, half (50.4%) underwent mastectomy and 42.0% underwent breast-conserving surgery. Although there was a trend towards more breast-conserving surgery over time, this trend did not reach statistical significantly (*P* = 0.07). For all ages, adjuvant radiotherapy after breast-conserving surgery increased over the years (all *P* values < 0.001). In the last period (2003–2005) the highest percentage of women receiving radiotherapy was in the age 65–75 (82.4%) and the lowest in the elderly of 75 years and older (51.9%). Radiotherapy after mastectomy was performed in 3.4% for the patients younger than 50 years and increased over time from 2.0% to 5.6% (*P* = 0.004). In the other age groups, the proportion of patients undergoing radiotherapy after mastectomy was lower (2.2% for 50–64 years, 2.4% for 65–75, and 1.2% for the patients aged 75 years and older, resp.) and did not change significantly over time.

Table 3 shows the observed mortality, the expected mortality based on the general population, and the excess mortality as the ratio of the observed and expected mortality according to age and treatment (patients who received no surgery excluded). As radiotherapy after mastectomy was rarely given, mastectomy with or without radiotherapy were merged. In patients who underwent breast-conserving surgery (with or without radiotherapy) observed mortality did not significantly exceed expected mortality resulting in ratios around 1.0. For the younger patients who underwent mastectomy, a significant relative mortality was recorded (2.6; *P* < 0.001). For all strata of patients over the age of 50 years who underwent mastectomy, observed and expected mortality were close resulting in a relative mortality of around 1.0. Overall, all surgically treated patients aged 50 years and older experienced no excess mortality due to DCIS (ratio of 1.0). All ages combined, 704 deaths within 10 years were observed versus 692.1 expected based on the general population with a ratio of 1.0 expressing no excess mortality of DCIS in the Netherlands. 

## 4. Discussion

Due to the introduction of breast cancer screening, incidence of DCIS has increased dramatically in the last years. Despite the increasing incidence not many population-based reports are available that report mortality in this group of women. The present population-based study of almost 8500 patients in the Netherlands diagnosed between 1997–2005 shows that excess mortality was observed for patients younger than 50 years. However, no excess mortality in surgically treated women over 50 years with DCIS was observed. This means that after the diagnosis and treatment of DCIS the women experienced a similar mortality as age and year matched women in the general population.

### 4.1. Treatment

Understanding the care received by women with DCIS is important since it is highly curable, its incidence is rising (from 4.9 (1989) to 18.6 (2008) per 100 000), and it is often detected in otherwise healthy women and there is a possibility of overtreatment [9]. Overall, 45% of the women in the Netherlands underwent mastectomy for DCIS. In many such patients, mastectomy may have been medically appropriate, based on patient preferences or the underlying practice of individual surgeons or institutions [2]. 

Elderly 75 years or older did not undergo surgery in 7.6%. The reasons for this are unclear, but the result is probably explained by patients who are unfit for surgery due to many comorbidities or patient preferences [10]. Although mastectomy results in a cure rate approaching 100%, this may be overtreatment for some patients, particularly those with small, mammographically detected lesions [11]. Moreover, there are no randomized studies demonstrating that mastectomy is better than conservative surgery followed by radiotherapy for patients with DCIS [1]. The role of radiotherapy after breast-conserving surgery is supported by large randomized studies for improvement of local control; however, none of these studies showed an improvement in survival or decrease in the risk of distant metastases [1, 12–14]. A recent overview of the randomized trials of radiotherapy in DCIS showed that radiotherapy reduced the absolute 10-year risk of ipsilateral recurrent DCIS by 8.4% and of ipsilateral invasive cancer by 8.5% (both *P* < 0.00001) [15]. However, after 10 years of follow-up there was no significant effect on breast cancer mortality, mortality from causes other than breast cancer, or overall mortality. In the present study administration of adjuvant radiotherapy after breast-conserving surgery increased through the years for all ages, however, remained lower for the elderly aged 75 years and older. The identification of low risk groups within the elderly patients in whom radiotherapy can be omitted as well as the development of newer radiation techniques should be a priority [1].

### 4.2. Mortality

In the present study, data concerning the cause of deaths was not available. However, we were able to estimate the excess mortality by comparing the mortality in the cohort to mortality in the general population (matched for sex, year, and age). The present study showed no excess mortality as compared to the general population in patients who underwent surgery. Patients who were not surgically treated were excluded from this analysis as they are probably considered to frail and by such are not comparable to the general population. As far as we know, there is only one published population-based report of the likelihood of breast cancer death among women with DCIS (*n* = 7072) [3]. Breast cancer deaths were assessed in two groups based on the introduction of screening mammography (1978–1983 and 1984–1989). Among women diagnosed in the early period, 1.5% died of breast cancer within 5 years and 3.4% in 10 years; among the women diagnosed in the latter period 0.7% died of breast cancer within 5 years and 1.9% in 10 years. The study of Ernster et al. reported a 10-year standardized mortality ratio of 1.9 (95% CI 1.2–2.3). Direct comparison of the numbers is however not possible as the study of Ernster calculated the standardized mortality ratio and the present study the estimated excess (relative) mortality. The latest numbers in the study of Ernster et al. were from 1984–1989 while the present study describes 1997–2005. Diagnostic precision (by introduction of the digital mammography) has probably improved in that period so that patients are less likely to have unrecognized microinvasive breast cancer or the proportion of detected DCIS with low malignant potential has increased [3]. This could also possibly reduce the excess mortality due to breast cancer in our cohort as compared to Ernster et al. 

As almost all women were treated surgically, it is impossible to know from these data the extent to which the low excess mortality from breast cancer among women with DCIS results from effective treatment or reflects the relative benign nature of the disease or probably both [3]. Remarkably, we did find an excess mortality in the younger patients (<50 years) treated with mastectomy. It could be that a large proportion of this group are BRCA1/2 carriers; however we have no information to verify this. Mortality was stratified for age (to assess differences in young and elderly patients) and treatment. The aim of the stratification in treatments was not to compare treatments between strata as this would not be possible due to confounding by indication. Instead, we aimed to assess excess mortality over strata for each surgical treatment group. In the present study we had no data concerning recurrences in individual patients. However, despite the fact that some of these women would have experienced a recurrence, excess mortality due to that recurrence is presumably low. Approximately half of the recurrences are not invasive and can be cured with additional surgery [1]. Furthermore, patients who present with invasive cancer have also a low risk of distant disease [1]. 

For some ages excess mortality was below 1.0 (observed mortality did not exceed expected mortality) indicating that women with DCIS represent a generally healthy subgroup of the population which was also confirmed in the study of Ernster et al. [3]. Women who present for mammography may have healthier lifestyles than other women; studies have shown that women who undergo regular screening are more socioeconomically advantaged and practice more preventive health behaviors than women who do not [3, 16, 17]. Moreover, breast cancer is more often diagnosed in women with a higher socioeconomic background. 

In conclusion, the present population-based study of almost 8500 patients in the Netherlands shows no excess mortality in surgically treated women over 50 years with DCIS; observed and expected mortality were almost equal resulting in a relative mortality due to the DCIS of 1.0. 

## Figures and Tables

**Table 1:** Characteristics of the population with ductal carcinoma *in situ* (DCIS) 1997–2005.

Variable		Age (years)				
		<50	50–64*	65–75*	>75	Total
		*N* (%)	*N* (%)	*N* (%)	*N* (%)	*N* (%)

Period	1997–1999	546 (33.2)	1196 (29.1)	529 (27.0)	232 (32.3)	2503 (29.7)
	2000–2002	511 (31.1)	1327 (32.5)	723 (36.9)	245 (34.1)	2806 (33.3)
	2003–2005	587 (35.7)	1574 (38.4)	709 (36.1)	242 (33.6)	3112 (37.0)
Grade	I	220 (13.4)	482 (11.8)	278 (14.2)	120 (16.7)	1100 (13.1)
	II	391 (23.8)	987 (24.1)	507 (25.8)	146 (20.3)	2031 (24.1)
	III	644 (39.2)	1726 (42.1)	712 (36.3)	177 (24.6)	3259 (38.7)
	Unknown	389 (23.6)	902 (22.0)	464 (23.7)	276 (38.4)	2031 (24.1)

Total		1644	4097	1961	719	8421

*Invited for mass screening program in the Netherlands.

**Table:** (a) Surgical treatment for DCIS over time in the Netherlands. (b) Adjuvant radiotherapy, over time, for patients with DCIS undergoing breast conserving surgery (BCS) or mastectomy

Age		1997–1999	2000–2002	2003–2005	*P* value (years)
Distribution of type of surgery over time (%)					

**<50**	BCS	38.9	46.3	43.6	0.4
	MAST	60.5	53.1	55.7	
	No surgery	0.6	0.6	0.7	
**50–64**	BCS	50.6	56.4	60.7	**<0.001**
	MAST	48.6	43.4	38.2	
	No surgery	0.8	0.2	1.1	
**65–74 **	BCS	50.3	52.6	62.6	**<0.001**
	MAST	48.8	46.2	36.8	
	No surgery	0.9	1.2	0.6	
**≥75**	BCS	37.1	43.4	44.8	0.07
	MAST	55.6	50.9	45.3	
	No surgery	7.3	5.7	9.9	

**Table:** (b)

Age	1997–1999	2000–2002	2003–2005	*P* value (years)
Breast-conserving surgery (radiotherapy %)				

**<50**	52.3	59.0	71.7	**<0.001**
**50–64**	46.4	69.3	81.6	**<0.001**
**65–75**	41.9	60.0	82.4	**<0.001**
**>75**	26.3	36.4	51.9	**<0.001**

Mastectomy (radiotherapy %)				

**<50**	2.0	2.3	5.6	**0.004**
**50–64**	1.8	3.0	1.9	0.8
**65–75**	1.4	4.1	1.2	0.6
**>75**	0	1.7	1.9	0.2

**Table 3:** Mortality (10 years) of the surgically treated patients with DCIS as compared to the general population according to age, stratified by treatment.

Treatment	Mortality	<50 years	50–64 years	65–75 years	>75 years	Overall
BCS+RT	Observed	7	34	52	24	117
	Expected	5.2	47.5	67.4	26.9	147
	Relative mortality	1.4	0.7	0.8	0.9	0.8*
BCS-RT	Observed	6	36	57	71	170
	Expected	3.8	29.4	49.6	74.5	157.3
	Relative mortality	1.6	1.2	1.1	1.0	1.1
MAST	Observed	29	61	100	119	309
	Expected	11.1	63.3	102.2	131.0	307.5
	Relative mortality	2.6***	1.0	1.0	0.9	1.0
Overall	Observed	44	156	233	271	704
	Expected	21.6	154.6	243.7	272.2	692.1
	Relative mortality	2.0***	1.0	1.0	1.0	1.0

*P < 0.05, **P < 0.01, ***P < 0.001. Median follow-up: 5.9 years (range 1.2–10.9).

## References

[1] Estevez LG, Alvarez I, Segui MA (2010). Current perspectives of treatment of ductal carcinoma in situ. *Cancer Treatment Reviews*.

[2] Baxter NN, Virnig BA, Durham SB, Tuttle TM (2004). Trends in the treatment of ductal carcinoma in situ of the breast. *Journal of the National Cancer Institute*.

[3] Ernster VL, Barclay J, Kerlikowske K, Wilkie H, Ballard-Barbash R (2000). Mortality among women with ductal carcinoma in situ of the breast in the population-based surveillance, epidemiology and end results program. *Archives of Internal Medicine*.

[4] Katz SJ, Lantz PM, Zemencuk JK (2001). Correlates of surgical treatment type for women with noninvasive and invasive breast cancer. *Journal of Women’s Health and Gender-Based Medicine*.

[5] Fisher ES, Welch HG (1999). Avoiding the unintended consequences of growth in medical care: how might more be worse?. *Journal of the American Medical Association*.

[6] Morrow M (2004). The certainties and the uncertainties of ductal carcinoma in situ. *Journal of the National Cancer Institute*.

[7] Kricker A, Armstrong B (2004). Surgery and outcomes of ductal carcinoma in situ of the breast: a population-based study in Australia. *European Journal of Cancer*.

[8] Ernster VL, Ballard-Barbash R, Barlow WE (2002). Detection of ductal carcinoma in situ in women undergoing screening mammography. *Journal of the National Cancer Institute*.

[9] Rakovitch E (2000). Part I. Epidemiology of ductal carcinoma in situ.. *Current Problems in Cancer*.

[10] Louwman WJ, Vulto JCM, Verhoeven RHA, Nieuwenhuijzen GAP, Coebergh JWW, Voogd AC (2007). Clinical epidemiology of breast cancer in the elderly. *European Journal of Cancer*.

[11] Morrow M, Strom EA, Bassett LW (2002). Standard for the management of ductal carcinoma in situ of the breast (DCIS). *Ca-A Cancer Journal for Clinicians*.

[12] Fisher B, Costantino J, Redmond C (1993). Lumpectomy compared with lumpectomy and radiation therapy for the treatment of intraductal breast cancer. *The New England Journal of Medicine*.

[13] Julien JP, Bijker N, Fentiman IS (2000). Radiotherapy in breast-conserving treatment for ductal carcinoma in situ: first results of the EORTC randomised phase III trial 10853. EORTC Breast Cancer Cooperative Group and EORTC Radiotherapy Group. *The Lancet*.

[14] Polgar C, Kahan Z, Orosz Z (2008). The role of radiotherapy in the conservative treatment of ductal carcinoma in situ of the breast. *Pathology and Oncology Research*.

[15] Early Breast Cancer Trialists’ Collaborative Group (EBCTCG), Correa C, McGale P (2010). Overview of the randomized trials of radiotherapy in ductal carcinoma in situ of the breast. *Journal of the National Cancer Institute. Monographs*.

[16] Lee JRJ, Vogel VG (1995). Who uses screening mammography regularly?. *Cancer Epidemiology Biomarkers and Prevention*.

[17] Hofer TP, Katz SJ (1996). Healthy behaviors among women in the United States and Ontario: the effect on use of preventive care. *American Journal of Public Health*.

